# *Ascaris lumbricoides* Infection Following School-Based Deworming in Western Kenya: Assessing the Role of Pupils' School and Home Water, Sanitation, and Hygiene Exposures

**DOI:** 10.4269/ajtmh.15-0362

**Published:** 2016-05-04

**Authors:** Joshua V. Garn, Charles S. Mwandawiro, Birgit Nikolay, Carolyn D. Drews-Botsch, Jimmy H. Kihara, Simon J. Brooker, Elses W. Simiyu, Collins Okoyo, Matthew C. Freeman

**Affiliations:** Department of Epidemiology, Rollins School of Public Health–Laney Graduate School, Emory University, Atlanta, Georgia; Eastern and Southern Africa Centre of International Parasite Control (ESACIPAC), Kenya Medical Research Institute (KEMRI), Nairobi, Kenya; Faculty of Infectious and Tropical Diseases, London School of Hygiene and Tropical Medicine, London, United Kingdom; Vector-borne Disease Control Unit, Ministry of Health, Nairobi, Kenya; Department of Environmental Health, Rollins School of Public Health, Emory University, Atlanta, Georgia

## Abstract

Water, sanitation, and hygiene (WaSH) technologies and behaviors can prevent infection by soil-transmitted helminth species independently, but may also interact in complex ways. However, these interactions are poorly understood. The purpose of this study was to characterize how school and home WaSH exposures were associated with *Ascaris lumbricoides* infection and to identify relevant interactions between separate WaSH technologies and behaviors. A study was conducted among 4,404 children attending 51 primary schools in western Kenya. We used multivariable mixed effects logistic regression to characterize how various WaSH exposures were associated with *A. lumbricoides* infection after annual school-based deworming. Few WaSH behaviors and technologies were independently associated with *A. lumbricoides* infection. However, by considering relevant interdependencies between variables, important associations were elucidated. The association between handwashing and *A. lumbricoides* depended largely upon the pupils' access to an improved water source. Among pupils who had access to improved water sources, *A. lumbricoides* prevalence was lower for those who handwashed both at school and home compared with neither place (odds ratio: 0.38, 95% confidence interval: 0.18–0.83; *P* = 0.01). This study contributes to a further understanding of the impact of WaSH on *A. lumbricoides* infection and shows the importance of accounting for interactions between WaSH technologies and behaviors.

## Introduction

It has been estimated that more than 1.45 billion people throughout the world are infected with soil-transmitted helminths (STHs), primarily roundworm (*Ascaris lumbricoides*), whipworm (*Trichuris trichiura*), and hookworms (*Necator americanus* or *Ancylostoma duodenale*).^1^ STH infections can lead to anemia,^2^ and slowed physical and cognitive development.^3^ School-aged children bear much of the burden of STH morbidity,^4^ which accounts for over 5 million disability-adjusted life years annually.^5^

Mass drug administration (MDA) programs that administer anthelminthic drugs, principally albendazole or mebendazole, at either the school or community level^6^ are being implemented throughout the world to reduce the prevalence of STHs and their associated morbidity.^7^,^8^ Although MDA greatly reduces parasite loads, deworming does not prevent transmission or reinfection.^9^ MDA efficacy varies depending on worm species and the type of deworming drug being used,^10^ but even when cure rates are high, the prevalence of STHs often return to near pretreatment levels within 6 months due to new infections.^11^

STH infection occurs most frequently through ingestion of eggs that were excreted via fecal material in the environment or in the case of hookworm directly through penetration of the skin by filariform larvae. As such, several studies have shown that transmission is preventable through improvement of environmental conditions and hygienic behaviors, specifically access to microbiologically safe water, improved sanitation, and handwashing with soap (WaSH).^12^–^15^ Although preventive effects of WaSH on STH infection have generally been observed, there is noted heterogeneity across studies, with both a diversity of previous study designs and a variety of evaluated WaSH behaviors and technologies.^12^–^15^

Characterizing the relationship between WaSH and STH infection is important, although it presents some methodological complexities in epidemiologic studies. First, WaSH is a multifaceted exposure containing several primary domains (e.g., water, sanitation, and hygiene), each of which is composed of various technologies and behaviors that vary between the school and home environments. Most prior WaSH studies have not attempted to model individual WaSH technologies and behaviors simultaneously in the multilevel school and home contexts in which they actually exist. Further, although some WaSH technologies and behaviors have the potential to be individually important, many are likely interdependent and interact in complex pathways to impact pathogen exposure (e.g., a pupil's handwashing behavior depends on soap and water availability). Some work has been done to characterize important interactions between WaSH services, but almost exclusively with diarrhea as the outcome.^16^–^20^ STHs have a different mechanism of transmission than diarrhea, and so characterizing these interactions for STHs may be equally important. We were only able to find one study where the explicit goal to assess WaSH interactions with STHs as the outcome.^21^

This analysis uses data from the third year of an ongoing monitoring and evaluation program (M&E) led by The Kenya Medical Research Institute (KEMRI), which used repeated cross-sectional surveys to assess the impact of yearly deworming on the prevalence of STHs in school children.^22^ The objectives of our particular study were to characterize how pupils' school and home WaSH exposures were associated with *A. lumbricoides* infection, and specifically to characterize how combinations of WaSH behaviors and technologies were associated with helminth infection. This study will facilitate an understanding of which individual and combinations of WaSH technologies and behaviors are most likely to reduce exposure to infective eggs and to prevent *A. lumbricoides* infection after MDA in control programs.

## Methods

### Study context.

The data used in these analyses come from an ongoing M&E of the Kenyan National School Based Deworming Program, where albendazole was provided annually to schoolchildren in efforts to reduce the overall prevalence of STHs and their associated morbidity.^22^ Two hundred schools were randomly selected from 20 districts from western Kenya in which STHs were endemic, and all of these schools would undergo long-term follow-up. Of these 200 schools, 70 schools were randomly selected to undergo further monitoring, where they would undergo more extensive surveillance that included the collection of pupil-reported WaSH conditions. Further details on the M&E design and sampling of districts are described elsewhere.^22^–^24^

### Study population.

Our research takes place among 51 of the 70 schools that collected pupil-reported WaSH conditions. Because of logistical delays in implementing the deworming program in this area, 19 monitored schools from Coast Province were excluded from our study. At each school visit, approximately nine boys and nine girls were randomly sampled from each grade (2–6) using random number tables, and individual exposure and outcome data were collected. A total of 4,404 pupils were surveyed, with an equivalent proportion of girls and boys (50%). These pupils were sampled and weighted to represent the 15,960 total enrolled pupils from grades 2–6.

### Data collection and follow-up timeline.

At each of the annual follow-ups, enumerators observed school WaSH conditions and collected pupils' reported WaSH histories. Stool samples were collected (both pre- and post-deworming), prepared on two separate slides, and the slides were analyzed independently for the presence of STH species using the Kato-Katz method.^25^ Data presented in this study were collected between May and June 2014, during the third year of the M&E, which took place 2 years after baseline (2012) and 1 year after the second mass deworming (2013). The deworming in this study was administered by the Ministry of Health.

The survey instruments were based on tools developed as part of a school-based WaSH trial previously administered in Nyanza Province, Kenya,^15^ and included a pupil survey to ascertain pupils' access to and use of different WaSH technologies and behaviors both at school and at home and a school survey to collect both teacher-reported and observed school WaSH conditions. All school and pupil surveys from the 2014 follow-up were collected by enumerators using Open Data Kit for Android-based smartphones (https://opendatakit.org/), and all surveys were conducted in the pupils' native language(s) by trained KEMRI staff.

### Outcome.

The outcome of interest for this study was infection with *A. lumbricoides* (yes versus no), as evidenced by *A. lumbricoides* eggs found in the pupil's stool sample.

We focused solely on the *A. lumbricoides* worm for several reasons. First, a higher prevalence of *A. lumbricoides* (17%) provided a higher powered analysis, whereas the prevalence of hookworm and *T. trichiura* were low (2% and 5%, respectively) and the adjusted models often had difficulty in converging. Second, albendazole is known to be more effective in the elimination of *A. lumbricoides* than either *T. trichiura* or hookworm,^10^ allowing us to more closely approximate cumulative incidence since the previous deworming. A final reason to focus on *A. lumbricoides* is that progress toward eliminating this worm might depend more heavily on WaSH because of the long infective period of *A. lumbricoides* eggs in soil.^26^ For example, recent study analyses of 153 schools participating in the overall M&E showed marked decreases in hookworm (from 15% to 2%) after two cycles of mass deworming, but the *A. lumbricoides* prevalence has only changed from 23% in 2012 to 15% in 2014.^24^

### Exposures.

Our primary exposures of interest were access to an improved water source, access to comprehensive sanitation (captured by several variables), and practice of handwashing with separate variables for each of these primary exposures at both school and home. Sometimes separate variables measured similar constructs, and in the Supplemental Appendix 1, we show correlations between these variables and reasoning why we included specific variables in our models. When two variables measured similar constructs, we used what we thought was the more objective variable for our models, but we also performed sensitivity analyses substituting the less-preferred variable to ascertain the impact of choosing one variable over another.

We observed the water source at each school and categorized these sources as improved or unimproved as defined by the World Health Organization (WHO)/United Nations Children's Fund Joint Monitoring Program (JMP) for Water Supply and Sanitation.^27^ Because water availability was so variable at schools, we further constrained our definition of an improved school water source by whether water was reliably available throughout the year, with water availability being teacher reported. The pupil's home water source was self-reported and was then categorized as either improved or unimproved as defined by the JMP.

We captured school and home sanitation characteristics with a number of different variables. We observed whether that pupil's school had met the WHO pupil to latrine ratio recommendations for each sex of pupils (< 25:1 for girls and < 50:1 + one urinal for boys).^28^ Enumerators also observed the percentage of latrines at the school that were VIP/waterborne, the presence of visible feces inside sanitation facilities (percentage of all school latrines with visible feces), and the presence of visible feces outside the sanitation facilities at the school (yes versus no). Access to home sanitation was pupil reported and was categorized as either having a personal sanitation facility in their compound, having a shared facility with other households, or not having access to a toilet facility at home.

Both school and home handwashing were assessed by self-report, and we compared pupils who reported always washing their hands after defecation to pupils who reported washing their hands only sometimes or never.

We also had interest in a number of other WaSH technologies and behaviors. Individual or home-level factors included the pupil-reported type of anal cleansing materials used (water, paper products, and leaves/rocks/nothing), pupil-reported floor type at home (earth versus other), pupil's shoe wearing as observed by the enumerator during the visit (closed shoe, sandal, and no shoes), and pupil's reported practice of eating soil (yes versus no)—a practice common in some areas of Kenya.^29^ Other WaSH variables that were collected but not included in our fully adjusted models are described in Supplemental Appendix 1.

We had originally considered the possibility of herd protection from some variables, including school handwashing, school sanitation, and community sanitation. That is, we consider the possibility that pupils' *A. lumbricoides* infection may be affected through group-level adherence, even in the absence of individual-level adherence.^30^,^31^ However, in each case, low heterogeneity of these aggregated school-level variables prevented inclusion of these variables in the model (Supplemental Appendix 1).

### Confounders.

To control for confounders of WaSH on *A. lumbricoides* infection, we included each of the following environmental and demographic variables in the models. Environmental variables included mean annual temperature, mean annual precipitation (both were linked to school locations from http://www.worldclim.org/bioclim), and the former province (under the new constitution, provinces no longer exist) where the schools were located (i.e., western Rift Valley and Nyanza Province). Demographic variables and other risk factors included the pupil's sex, grade, whether the pupil had siblings under the age of 5 years at home, and the pupil's socioeconomic status (using a continuous wealth index score constructed using principal component analysis).^32^ Variables included in the principal component analysis included household wall and roof type, having household electricity, and the ownership of various assets including a sofa, television, radio, bicycle, motorbike, car, or cell phone.

### Interaction specification.

We had interest in how combinations of WaSH behaviors and technologies were associated with helminth infection. We determined a priori a number of biologically plausible interactions of interest with public health relevance as shown in Table 1. We assessed multiplicative interaction using a holistic approach that first identified potential effect modifiers and their hypothesized direction of impact on other variables (based on a priori biological knowledge). We then used forward selection to identify if these a priori effect modifiers produced odds ratios (ORs) that were meaningfully different between groups (i.e., estimates in opposite directions or one null and the other not). Although our modeling strategy did not assess interaction based on statistical significance, post hoc analyses showed that the final interaction terms chosen for inclusion based on meaningful differences were also those same terms that had the smallest *P* values. When considering the inclusion of each interaction term, multicollinearity between terms (the presence of high condition indices with several high variance decomposition proportions)^33^ and model convergence were also factors used to determine whether each term could be included in the model.

### School and home WaSH together.

We jointly characterize our primary WaSH exposures in both school and home environments together. Specifically, we produced the OR for having access to an improved water source both at school and home together for handwashing and for having all of the ideal sanitation conditions (i.e., a personal toilet at home, all VIP latrines at school, no visible feces on school grounds, no visible feces in school latrines, and a school pupil to latrine ratio that meets the WHO recommendations).

### Data analysis and modeling strategy.

For the descriptive statistics, we accounted for the stratified random sampling, clustering of pupils within schools, and the sample weights to present percentages that were representative of all pupils in grades 2–6 from these schools. These descriptive statistics were carried out in SAS-Callable SUDAAN version 11.0.1. All of our unadjusted and multivariable analyses were carried out in SAS version 9.4 (Cary, NC).

We used multilevel mixed effects logistic regression models to quantify the relationship between individual WaSH technologies and behaviors, and the presence of an *A. lumbricoides* infection (yes versus no). We used multivariable models to account for WaSH variables and confounders simultaneously, first in a model without interaction terms. We then used multivariable models to account for WaSH variables, confounders, and interaction terms simultaneously, choosing the interaction terms as discussed above. The final model resembled the form:

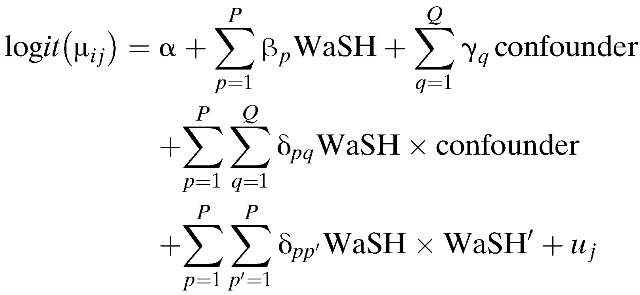


where μ_*ij*_ represents the probability of *A. lumbricoides* infection in the *i*th student within the *j*th school. The WaSH, confounder, and interaction coefficients are represented by β, γ, and δ, respectively. The subscript *p* indexes each of the various WaSH variables and the subscript *q* indexes each of the confounder variables so that there are *P* different WaSH variables overall and *Q* different confounding variables. The WaSH × confounder terms capture interactions between the *p*th WaSH variable and the *q*th confounding variable, and the WaSH × WaSH′ terms capture interactions between the *p*th WaSH variable and the *p*′th WaSH variable (where *p ≠ p*′). The WaSH variables were both individual-level variables (*ij*), and school-level variables (*j*), but subscripts *i* and *j* have been suppressed for simplicity. A random intercept *u*_*j*_ is included to account for clustering within the *j*th school.

The models were used to produce adjusted OR estimates for each separate WaSH variable of interest. We also used these same models to contrast groups of relevant WaSH covariates, for example, computing an OR that compares a linear combination of several covariates in the numerator to a different combination of covariates in the denominator. This has practical applications when one has either a significant interaction between two variables or when one has interest in simply characterizing a “joint effect” for a complex exposure (e.g., when similar WaSH variables exist in both school and home environments).

### Ethical approval.

Ethical approval was obtained by the KEMRI ethics committee (SSC protocol no. 2206). We obtained consent from the school committee and also from parents of pupils participating in the study. Parents/guardians were free to refuse participation of their children in the study. On the day of the school visit, the enumerators informed all children that their participation was voluntary and that they could opt out of the testing at any time—a practice considered to be ethical and practical in low-risk studies and interventions.

## Results

### WaSH conditions.

The observed WaSH conditions were substandard^28^ in many schools. Around half of the schools (49%) had handwashing facilities near the toilets, but only 12% of the schools had soap available at the handwashing facilities (Table 2). Regarding water access at school, 53% of schools had an improved water source and 57% had drinking water reliably available all year round; 20% of the schools had an improved water source that also provides water year round. Observations of sanitation facilities showed that 16% of the schools met the WHO pupil to latrine standards for girls and 26% met the WHO pupil to latrine standards for boys and that 39% of the schools had solely VIP/waterborne latrines.

The pupil-reported WaSH conditions were also substandard. Pupils reported always washing their hands with soap after defecation only 4% of the time while at school and 8% of the time while at home (Table 3). Just over half of pupils reported having an improved water source (51%) and a personal latrine in their compound (55%).

### *A. lumbricoides* prevalence.

The *A. lumbricoides* prevalence among pupils attending the 51 schools was 17% (95% confidence interval [CI]: 16–18%) 1 year after the second deworming round. This is compared with the baseline survey in 2012 when the *A. lumbricoides* overall prevalence was 24% (95% CI: 23–25%) in the same schools (unpublished data). The school intraclass correlation coefficient was 0.28 at follow-up.

### Deworming treatments.

Children were asked if they had received deworming treatments in the last year, and 89.8% reported that they had, and of those, 99.7% reported receiving those treatments in school (implying it was by the program). We asked head teachers at schools if they had been participating in deworming programs and who administered those deworming programs, and all head teachers indicated receiving deworming through the Ministry of Health (implying it was done by the program).

### WaSH and *A. lumbricoides* infection.

The unadjusted associations for *A. lumbricoides* (also for *T. trichiura* and hookworm) is shown in the Supplemental Appendix 2. Results from the adjusted analyses are shown in Table 4. In the adjusted model with no interaction terms, all of the estimates between our primary WaSH exposures of interest and *A. lumbricoides* infection had 95% CIs that spanned one. Specifically, the OR for handwashing at school was 0.65 (95% CI: 0.37–1.13; *P* = 0.14) and at home was 1.00 (95% CI: 0.71–1.39; *P* = 0.98) and the OR for having access to an improved water source at school was 1.44 (95% CI: 0.70–2.96; *P* = 0.32) and at home was 1.06 (95% CI: 0.84–1.32; *P* = 0.63). The sanitation estimates were particularly imprecise, with no consistent relationship across variables.

Of our secondary WaSH exposures of interest, shoe wearing was associated with lower *A. lumbricoides* infection, whereas anal cleansing at school, anal cleansing at home, floor type, and geophagy were not associated with *A. lumbricoides* infection. We also report the associations between *A. lumbricoides* infection and several non-WaSH covariates that are sometimes of interest in the wider literature. We observed that male pupils were more likely than female pupils to have an *A. lumbricoides* infection (OR: 1.33, 95% CI: 1.11–1.59; *P* < 0.01) and that pupils in younger grades were more likely to have an *A. lumbricoides* infection than pupils in grade 6 (grade 2 OR: 1.36, 95% CI: 1.03–1.80; grade 3 OR: 1.27, 95% CI: 0.95–1.68; *P* = 0.03; grade 4 OR: 1.18, 95% CI: 0.89–1.57; *P* = 0.26, grade 5 OR: 1.11, 95% CI: 0.84–1.47).

We explored the data for variable interactions among a number of a priori potential interaction terms (Table 1). Our final model included interaction terms between handwashing and having access to an improved water source, both at school and at home (Table 5). In the final interaction model, pupils' handwashing at school was associated with lower *A. lumbricoides* infection in schools that had an improved water source that reliably supplied water, (OR: 0.45, 95% CI: 0.23–0.89; *P* = 0.02), but not in schools with an unimproved water source (OR: 1.99, 95% CI: 0.73–5.37; *P* = 0.18, *P* interaction = 0.01). The interaction between handwashing and having an improved water source was less pronounced at home (*P* interaction = 0.29), at least when assessing this interaction using these main analysis variables. However, handwashing and the type of water source were measured in multiple ways, so we performed sensitivity analyses to assess the robustness of these associations and found that the interactions between handwashing and having an improved water source often persisted regardless of the variable we used in both the school and the home environments, although individual ORs varied (see Supplemental Appendix 3).

We contrasted relevant linear combinations of both the school and the home WaSH covariates for each of the three WaSH domains (Table 6), also accounting for the interactions we found between handwashing and having access to an improved water source. The OR for handwashing at both school and home compared with neither place was 0.38 (95% CI: 0.18–0.83; *P* = 0.01) among pupils that also had access to an improved water source and was 2.34 (95% CI: 0.78–7.01; *P* = 0.13) among pupils that did not have access to an improved water source. The OR for having access to an improved water source at both school and home compared with neither place was 0.26 (95% CI: 0.059–1.17; *P* = 0.08) among pupils that always handwashed and was 1.63 (95% CI: 0.76–3.46; *P* = 0.20) among pupils that did not report handwashing. The OR for having a personal toilet at home, all VIP latrines at school, no visible feces on school grounds, no visible feces in school latrines, and a school pupil to latrine ratio that meets the WHO recommendations compared with having none of these was 0.93 (95% CI: 0.22–4.02; *P* = 0.92).

## Discussion

This study is one of the first to assess the association between *A. lumbricoides* infection and a wide variety of WaSH technologies and behaviors practiced by school pupils. The study demonstrates that some WaSH behaviors and technologies are interdependent upon combinations of WaSH variables. For example, the association between handwashing and *A. lumbricoides* depended upon the school's access to an improved water source that reliably supplied water. We also found strong preventive estimates when we considered handwashing both at school and at home together, compared with at neither place. However, for many of the WaSH variables, we did not observe clear patterns between WaSH and *A. lumbricoides* infection.

Our findings suggest that, a school's access to an improved water source is important for the success of handwashing interventions. Our models had the capacity to capture the effects of WaSH simultaneously at school and at home, and we observed an especially strong association between handwashing and *A. lumbricoides*, but again depending on presence of an improved water source both at school and at home. These results may shed light on the results from a recent study in Kenya, which found reductions in enrollment and diarrheal illness but only in those schools that were also provided a water source.^34^ Other school WaSH studies, including meta-analyses, often consider either water or sanitation or hygiene without considering their codependence,^12^,^13^ but this may overlook valuable information. Another hypothesis for why we might have observed this interaction between handwashing and an improved water source, may have little to do with water quality. It is possible that some pupils did not truthfully respond about handwashing behavior and that by including this interaction term, pupils who reported always handwashing but sometimes lacked the capacity to do so would be moved into a separate “stratum” from those individuals who reported always handwashing and also had the capacity to do so, allowing the handwashing estimates to differ by differing levels of adherence. Other handwashing variable constructs that we used in sensitivity analyses showed similar results, indicating robustness across measures. Although our findings from our interaction model—that handwashing requires water—are seemingly obvious, the codependence of these separate WaSH domains is an important message when trying to implement handwashing worldwide.

Even though we did not observe other pre-hypothesized interactions in this population, there may still be merit to assessing these interactions in other populations. One possibility for why we did not observe more interactions is that our analyses may have only been adequately powered to detect the strongest interactions, and weaker interactions may have been overlooked. More pupils who practiced WaSH would have improved the power of our analyses. It is also possible that these interactions simply do not exist in this population or that they exist on the additive scale.

Meta-analyses, primarily from non-school settings, have found decreased STH infection with improved sanitation access.^12^,^13^ A potential message from our article is that the definition that one uses for sanitation matters. We observed that the sanitation variables that were more closely tied to reducing fecal exposure, such as whether the latrines were VIP, were also more likely to be associated with lower *A. lumbricoides* infection. One possibility for our finding of higher *A. lumbricoides* infection among pupils in schools that met the WHO pupil to latrine ratio guidelines is that increased use of dirty latrines may increase pupils' exposure to disease.^35^ A lower pupil to latrine ratio has been found to be associated with increased latrine use.^36^ Other studies that have found latrine provisions to be associated with increased pupil hand contamination^37^ or have found associations between dirty latrines and bacterial pathogens throughout the bathroom,^38^ diarrhea,^35^ vomiting,^35^ and dysentery.^39^ However, we assessed the interaction between this latrine access variable and latrine cleanliness and did not find a meaningful interaction. The observation of marginally increased *A. lumbricoides* infection among pupils with better latrine access adds to evidence that simply meeting international coverage targets, in the absence of uptake or of a reduction in exposures, may be insufficient to improve health.^40^,^41^

A previous school-based STH reinfection study by Gass and others^21^ used two recursive partitioning methodologies (i.e., classification and regression trees and conditional inference trees) to identify various WaSH interactions. The interactions that were identified in their study differed by methodology and were often “counterintuitive.” Our approach identified fewer interactions overall and more intuitive interactions, but this was probably in part because we built our models and included potential interactions based largely on a priori biological plausibility. Recursive partitioning methodologies may be better for hypothesis generation,^21^ whereas our approach may be better when there is an interest in causal inference.

Shoe wearing was strongly associated with *A. lumbricoides* infection in each analysis, and floor type was associated with *A. lumbricoides* in the unadjusted analysis. These may work through a common mechanism, although it is unclear how the eggs would be ingested. Shoe wearing has been associated with decreased STH infection in other studies, although usually with hookworm,^12^ as hookworm can be contracted through the skin. It is possible that the observed association between *A. lumbricoides* and shoe wearing is related to socioeconomic status, although we included variables that control for household wealth.

Our study emphasizes the role of WaSH in the context of school-based national deworming programs. Albendazole, which was used in the ongoing program, is known to have a high cure rate for *A. lumbricoides* (95%).^10^ Treatment coverage of the deworming program was also high (95%) in the 153 schools from the same provinces participating in the overall M&E.^24^ Taken together, this is suggestive that most of the observed *A. lumbricoides* infections in our study probably represent new infections since the previous deworming. School-level access and adherence to WaSH was substandard^28^ in many schools, and improving WaSH conditions may be an important component to preventing these new infections.

Our study used annual school-wide deworming and repeated cross-sectional assessments to approximate reinfection since the previous deworming. We call our outcome infection rather than “re”infection due to the possibility that some children may not have been successfully dewormed. We did not explicitly measure unprogramed deworming. Our results will be most generalizable to populations undergoing similar mass deworming programs.

There are several potential limitations of our study. The Kato-Katz assay has low sensitivity for the diagnosis of *A. lumbricoides* infection, especially in individuals with low intensity of infection.^42^ Such low intensity infections may be more common in settings where MDA had been delivered, leading to an underestimation of post-MDA *A. lumbricoides* prevalence. As with any observational study, there is the possibility of confounding by unknown variables, although we did control for known confounders including pupil's grade, sex, whether pupils had siblings under the age of 5 years, household wealth score, the mean annual temperature, annual precipitation, and province (along with all of our various WaSH variables of interest). Our WaSH exposures were primarily self-reported, although we were sometimes able to use structured observations to collect some of the variables. We also only used a single day of observations and a single survey to capture pupils' time-varying WaSH histories. We were able to calculate correlations between variables measuring similar constructs that also captured different time frames, and strong correlations between these different constructs suggest consistency in our measures (Supplemental Appendix 1). It is not clear if there were systematic reporting biases, but the low prevalence of several self-reported exposures, such as handwashing, suggests that overreporting of variables might have been rare. We were limited in that we did not have the ability to observe the sanitation conditions in the home environment and therefore were not able to include variables such as the contamination of the latrine at home. We only assessed multiplicative interaction, primarily because the log-binomial regression and modified Poisson regression models that we had originally intended to use to assess additive interaction did not converge. As our outcome was not rare, we were unable to use the OR to assess additive interaction. Future studies should also assess additive interaction, if possible. Also, as our outcome was not rare, the OR estimates are further from the null than the corresponding prevalence ratio estimates would have been had we instead been able to use Poisson or log-binomial models.

## Conclusions

Our study shows the importance of accounting for interdependencies between different WaSH technologies and behaviors in understanding the associations between STH and WaSH. When not accounting for important interactions, we found very few associations between WaSH behaviors and technologies and *A. lumbricoides* infection, but accounting for these interactions elucidated important associations. We observed that the association between handwashing and *A. lumbricoides* also depends upon the school having access to an improved water source that reliably supplied water. We also observed strong preventive estimates, when we considered adherence to handwashing at school and home together.

## Supplementary Material

Supplemental Datas.

## Figures and Tables

**Table 1:** Potential interactions of interest

Variable	Potential effect modification by	Retained*
Handwashing at school	Type of school water source†	Yes
Handwashing at home	Type of home water source†	Yes
Handwashing at school	Type of anal cleansing materials	No
Handwashing at home	Type of anal cleansing materials	No
Handwashing at home	Baseline worm prevalence	No
Handwashing at school	Baseline worm prevalence	No
The type of school water source†	Baseline worm prevalence	No
The type of home water source†	Baseline worm prevalence	No
Latrine access at home	Baseline worm prevalence	No
Latrine access at school	Baseline worm prevalence	No
Open defecation at home	Baseline worm prevalence	No
Visible feces in the open at school	Baseline worm prevalence	No
Visible feces in latrines at school	Baseline worm prevalence	No
Soil eating behavior	Baseline worm prevalence	No
Open defecation at home	Any of the climate variables	No
Visible feces in the open at school	Any of the climate variables	No
Visible feces in latrines at school	Any of the climate variables	No
Visible feces in the open at school	Shoe wearing	No
Visible feces in latrines at school	Shoe wearing	No
A natural floor at home	Shoe wearing	No
The interactions between separate school and home WaSH variables‡		No

WaSH = water, sanitation, and hygiene.

* All of these potential effect modifiers were assessed using forward selection, and only those effect modifiers that produced estimates in that were meaningfully different between groups were retained in the final model.

† Improved vs. unimproved, as defined by the World Health Organization/United Nations Children's Fund Joint Monitoring Program for Water Supply and Sanitation.^27^

‡ We assessed if there was multiplicative interaction between school and home environments for variables such as handwashing, the type of water source, and latrine access, which each had separate variables that captured the school and home environments.

**Table 2:** Observed and teacher-reported WaSH conditions at 51 Kenyan primary schools

	*N*	%
School hygiene		
Handwashing facilities near the toilets	25	49
Water in handwashing facilities	30	58
Soap available at the handwashing facilities	6	12
School water		
Improved water source for drinking*	27	53
Drinking water reliably available year round	29	57
Improved water source that reliably supplied water	10	20
School sanitation		
Meets the WHO pupil to latrine ratio standards for girls†	8	16
Meets the WHO pupil to latrine ratio standards for boys†	13	26
All latrines in school were VIP/waterborne	20	39
Latrines clean in school‡	11	22
Feces visible on grounds outside the latrines	16	31

WaSH = water, sanitation, and hygiene; WHO = World Health Organization.

* As defined by the WHO/United Nations Children's Fund Joint Monitoring Program for Water Supply and Sanitation.^27^

† There was one all-boys school and one all-girls school, so the denominator for this variable is 50 schools. The WHO pupil to latrine ratio recommendations are 25:1 for girls, and 50:1 + one urinal for boys.^28^

‡ No visible feces inside any of the latrines.

**Table 3:** Pupil-reported WaSH conditions by 4,404 respondents, weighted to represent 15,960 pupils from grades 2–6 in 51 Kenyan primary schools

	%*	SE*
School hygiene		
School provides a handwashing place	62.8	0.8
Water always available for handwashing at school	19.9	0.9
Soap always available for handwashing at school	1.0	0.2
Handwashed with soap and water the last time they defecated at school	12.3	0.8
Always handwashed with soap and water after defecating at school	3.8	0.4
School water		
Water always available for drinking at school	21.0	0.9
School sanitation		
Usually defecate in the latrine/toilet at school	99.4	0.1
Used a latrine/toilet at school last time they defecated at school	97.5	0.3
Think their friends always defecate in the latrine/toilet at school	75.7	1.0
Home hygiene		
Have a handwashing place at home	49.7	1.0
Water always available for handwashing at home	18.9	0.8
Soap always available for handwashing at home	10.3	0.6
Handwashed with soap and water the last time they defecated at home	33.1	1.0
Always handwashed with soap and water after defecating at home	8.1	0.5
Home water		
Have an improved water source for drinking† at home	50.7	1.08
Water always available for drinking at home	85.0	0.9
Home sanitation		
Have a personal toilet/latrine in your home/compound?	55.0	1.0
Have a shared toilet/latrine in your home/compound?	42.0	1.0
No toilet/latrine in your home/compound	2.9	0.3
Usually defecate in the latrine/toilet at home	98.6	0.2
Used a latrine/toilet at home last time they defecated at home	96.8	0.3

SE = standard error; WaSH = water, sanitation, and hygiene.

* Weighted % and SE accounted for the stratified random sampling, clustering of pupils within schools, and the sample weights.

† As defined by the World Health Organization/United Nations Children's Fund Joint Monitoring Program for Water Supply and Sanitation.^27^

**Table 4:** ORs comparing WaSH technologies and behaviors with *Ascaris lumbricoides* infection after school-based deworming among 4,404 pupils attending 51 Kenyan primary schools

	Adjusted model† (no interaction terms)		Adjusted model† (interaction terms)	
	OR (95% CI)	*P*	OR (95% CI)	*P*
School WaSH variables				
Always handwashed after defecation		0.14	Interaction	
Yes	0.65 (0.37–1.13)		See Table 5	
No	Referent			
Improved water source that reliably supplied water		0.32	Interaction	
Yes	1.44 (0.70–2.96)		See Table 5	
No	Referent			
Pupil to latrine ratio acceptable		0.05		0.05
Yes	1.58 (0.99–2.53)		1.58 (0.99–2.53)	
No	Referent		Referent	
Percent of latrines with visible feces on floor/walls		0.99		0.94
All latrines have feces	0.99 (0.28–3.49)		0.96 (0.27–3.39)	
No latrines have feces	Referent		Referent	
Percent of latrines that were VIP at school		0.48		0.49
All latrines were VIP	0.75 (0.33–1.68)		0.75 (0.33–1.69)	
No latrines were VIP	Referent		Referent	
Feces visible outside latrines		0.42		0.41
Yes	1.37 (0.74–2.18)		1.39 (0.64–3.04)	
No	Referent		Referent	
Anal cleansing with		0.45		0.45
Water	0.84 (0.42–1.69)		0.84 (0.42–1.69)	
Leaves/rocks/nothing	Referent		Referent	
Paper product	1.12 (0.87–1.44)		1.12 (0.87–1.44)	
Home WaSH variables				
Always handwashed after defecation		0.98	Interaction	
Yes	1.00 (0.71–1.39)		See Table 5	
No	Referent			
Improved water source		0.63	Interaction	
Yes	1.06 (0.84–1.32)		See Table 5	
No	Referent			
Toilet		0.78		0.75
Shared	1.08 (0.85–1.36)		1.08 (0.86–1.37)	
No toilet	0.96 (0.55–1.66)		0.96 (0.56–1.67)	
Personal	Referent		Referent	
Anal cleansing with		0.28		0.28
Water	1.62 (0.85–3.08)		1.54 (0.80–2.95)	
Leaves/rocks/nothing	Referent		Referent	
Paper product	0.98 (0.77–1.24)		0.98 (0.77–1.25)	
Other WaSH variables				
Shoe wearing		< 0.01		< 0.01
Closed shoes	0.67 (0.54–0.84)		0.67 (0.54–0.84)	
Sandals	0.62 (0.48–0.81)		0.62 (0.48–0.81)	
No shoes	Referent		Referent	
Type of floor in home		0.64		0.63
Earth/sand	1.08 (0.79–1.47)		1.08 (0.79–1.48)	
Cement/wood/iron sheets	Referent		Referent	
Student eats soil (geophagy)*		0.42		0.42
Yes	1.15 (0.82–1.60)		1.13 (0.81–1.57)	
No	Referent		Referent	
Data not shown for confounders†	Data not shown†		Data not shown†	

CI = confidence interval; OR = odds ratio; WaSH = water, sanitation, and hygiene.

* Geophagy is a soil eating practice common in some parts of Kenya.

† The adjusted model controlled for all of the variables in this table, and other confounders including pupil's grade, sex, whether pupils had siblings under the age of 5 years, household wealth score, the mean annual temperature, annual precipitation, and province. All models accounted for clustering of pupils within schools.

**Table 5:** ORs showing interaction between pupil handwashing and type of water source among 4,404 pupils attending 51 Kenyan primary schools

Interaction model*	Among those with improved water source†	Among those with unimproved water source	*P* assessing interaction
Always handwash at school			
Yes	0.45 (0.23–0.89); *P* = 0.02	1.99 (0.73–5.37); *P* = 0.18	*P* = 0.01
No	Referent	Referent	
Always handwash at home			
Yes	0.84 (0.52–1.35); *P* = 0.47	1.18 (0.76–1.84); *P* = 0.47	*P* = 0.29
No	Referent	Referent	
	Among those who always handwash	Among those who do not handwash	*P* assessing interaction
Improved water source at school†			
Yes	0.34 (0.09–1.32); *P* = 0.12	1.49 (0.72–3.08); *P* = 0.28	*P* = 0.01
No	Referent	Referent	
Improved water source at home			
Yes	0.77 (0.42–1.43); *P* = 0.41	1.09 (0.86–1.37); *P* = 0.48	*P* = 0.29
No	Referent	Referent	

OR = odds ratio; WaSH = water, sanitation, and hygiene.

* Models included handwashing × water interaction terms and controlled for all of the other WaSH variables and confounder variables.

† At many schools, improved school water sources did not reliably supply water throughout the year, so here we constrained the definition of an improved school water source to also require water reliability.

**Table 6:** ORs* jointly characterizing both school and home WaSH together on *Ascaris lumbricoides* infection among 4,404 pupils attending 51 Kenyan primary schools

Always handwashed	Among those with an improved WS†	Among those without an improved WS†
At both school and home	0.38 (0.18–0.83); *P* = 0.01	2.34 (0.78–7.01); *P* = 0.13
At neither place	Referent	Referent
Always had access to an improved WS*	Among those who always handwashed	Among those who did not handwash
At both school and home	0.26 (0.059–1.17); *P* = 0.08	1.63 (0.76–3.46); *P* = 0.20
At neither place	Referent	Referent
Comprehensive sanitation‡	Among everybody	
At both school and home	0.93 (0.22–4.02); *P* = 0.92	
At neither place	Referent	

OR = odds ratio; WaSH = water, sanitation, and hygiene; WS = water source.

* Uses the fully adjusted primary interaction model from Table 5.

† Improved WS that reliably supplied water.

‡ This compares a pupil with a personal toilet at home, all VIP latrines at school, no visible feces on school grounds, no visible feces in school latrines, and a school pupil to latrine ratio that meets the World Health Organization recommendations, to a pupil with none of these.
