# Supplementary material for: Ascaris lumbricoides Infection Following School-Based Deworming in Western Kenya: Assessing the Role of Pupils' School and Home Water, Sanitation, and Hygiene Exposures
Source: Am J Trop Med Hyg. 2016 May 4;94(5):1045–54. doi: 10.4269/ajtmh.15-0362 (PMC4856601; doi:10.4269/ajtmh.15-0362)
Supplement: Supplementary file 1 [file SD3.pdf]

SUPPLEMENTAL APPENDIX 1

VARIABLE SPECIFICATION PROCEDURES

Because there were many water, sanitation, and hygiene (WaSH) technologies and behaviors of interest, we used a number of guiding criterion during the variable specification and model specification.

We first aggregated variables to the correct levels based on causal hypotheses of how they might affect the outcome. Some variables were collected at the pupil level, although they were intrinsically school-level variables, and were therefore aggregated to the school level.

We assessed the homogeneity of variables to see if they have enough variation to be included in the analyses. We also assessed that there were sufficient numbers in cells of categorical variables, and when we observed small cell counts, we considered the possibility of combining similar categories to resolve the problem. We had originally considered the possibility of herd protection from some variables, including school handwashing, school sanitation, and community sanitation. That is, we allow for the possibility that pupils' *Ascaris lumbricoides* infection may be affected through group-level adherence, even in the absence of individual-level adherence. When considering the school-level handwashing variable, in 44 of the 51 schools, fewer than 10% of the pupils reported always washing their hands after defecation. For sanitation variables, almost all pupils reported always using a latrine for defecation at both school and home. Because heterogeneity was poor, we were unable to assess herd protection as originally intended.

Although access to WaSH is important, we generally assumed that pupils' helminth infection could be affected only through the use of WaSH and not through access alone. For example, the presence of a handwashing station can only affect pupil health through handwashing.

Our primary exposures of interest were access to an improved water source, access to comprehensive sanitation (captured by several variables), and practice of handwashing, with separate variables for each of these primary exposures at both school and at home. We also had interest in several other WaSH technologies and behaviors. We attempted to control for a number of important confounders, and to include relevant interactions between variables. The inclusion of each variable was chosen a priori based on biological plausibility and on the previous literature. Sometimes separate variables measured similar constructs, and in Supplemental Table 1 we show correlations between these variables, and the reasons why we chose to include specific variables in our models. We also assessed collinearity of variables in the full model and eliminated terms that were collinear (measured by the presence of high condition indices with several high variance decomposition proportions).[1] Further details on each variable of interest are also discussed in the article.

It was initially unclear whether or not we should control for the prevalence of *A. lumbricoides* at baseline, as we hypothesized that it could act as either an intermediate variable or a confounding variable. We therefore used a data-driven approach, and ran two separate adjusted models, with and without the "baseline *A. lumbricoides* prevalence" variable, comparing the change in the coefficients from the model for each of the WaSH variables between the reduced and full model. The two models gave generally similar results (both the point estimates and variances), indicating minimal confounding due to this variable. Only the estimate for one variable—the % of ventilated improved pit latrines in school—was > 10% different from the fully adjusted estimate, but we believed this was more likely to be an intermediate. For this reason, we do not control for the baseline prevalence of *A. lumbricoides* in our article.

REFERENCES

1. Kleinbaum DG, Klein M, 2010. *Logistic Regression: A Self-Learning Text*. New York, NY: Springer.
2. Vyas S, Kumaranayake L, 2006. Constructing socio-economic status indices: how to use principal components analysis. *Health Policy Plan 21:* 459–468.
3. Mokken RJ, 1971. *A Theory and Procedure of Scale Analysis: With Applications in Political Research*. The Hague, The Netherlands: Mouton.

Supplemental Table 1

Variable specification procedures

Variables	Type	Level	Included	Variable notes
School hygiene				
School provides a handwashing place	Pupil reported	Aggregated to school level	No, only adherence is relevant	Handwashing only functions through adherence, so having access alone was not important to our models. We considered a Mokken scale for all of these variables, and they were highly scalable: Loevinger's *H* coefficient = 0.70.* However, the scale overemphasized access and underemphasized actual adherence. The correlation coefficient between last handwashing and always handwashing was 0.45 ($P < 0.01$). We used the always handwashing variable in our primary analysis as it represented the public health ideal, but we used the last handwashing variable in a sensitivity analysis as it is less prone to recall bias†
Water always available at that place	Pupil reported	Aggregated to school level	No, only adherence is relevant	
Soap always available at that place	Pupil reported	Aggregated to school level	No, only adherence is relevant	
Handwashed with soap and water the last time they defecated	Pupil reported	Pupil level	No, redundant	
Always handwash with soap and water after defecating	Pupil reported	Pupil level	Yes	
Handwashed with soap and water the last time they defecated (same as above, but aggregated)	Pupil reported	Aggregated to school level	No, homogenous	We had originally considered the possibility of group-level, or herd protection. However, handwashing was poor at most schools (see Table 3), so the variable lacked the necessary heterogeneity to be able to include in our multivariable models
Always handwash with soap and water after defecating (same as above, but aggregated)	Pupil reported	Aggregated to school level	No, homogenous	
Handwashing facilities near the toilets	Observed	School level	No, only adherence to handwashing is relevant	We assumed that access to handwashing supplies could only improve health through actual use (i.e., washing ones hands). These variables assessing access alone were therefore not included in our models
Water in handwashing facilities	Observed	School level	No, only adherence to handwashing is relevant	
Soap available at the handwashing facilities	Observed	School level	No, only adherence to handwashing is relevant	
School water				
Water always available for drinking	Pupil reported	Aggregated to school level	No, redundant	Pupil-reported water availability and teacher-reported availability were measured with different questions; the correlation coefficient between these original continuous variables was 0.35 ($P < 0.01$). We believed the teacher reported value would be less prone to reporting errors, and so we used a categorized version of this variable (always available vs. not). For our primary analysis, we collapsed this variable with the improved water source variable. We used the other variables with similar constructs in sensitivity analyses‡
Improved water source for drinking‡	Observed	School level	No, redundant	
Drinking water is reliably available	Teacher reported	School level	No, redundant	
Improved water source that reliably supplied water	Multiple sources	School level	Yes	
School sanitation				
Usually defecate in the latrine/toilet at school	Pupil reported	Aggregated to school level	No, homogenous, redundant	The construct we wanted to measure was contamination by open defecation at the school. Many of these variables were too homogenous to use. We used the observed variable (i.e., whether there were feces visible on the grounds), as it was sufficiently heterogeneous and was the most direct measure
Used a latrine/toilet at school last time they defecated	Pupil reported	Aggregated to school level	No, homogenous, redundant	
Think their friends always defecate in the latrine/toilet at school	Pupil reported	Aggregated to school level	No, redundant	
Feces visible on grounds outside the latrines	Observed	School level	Yes	

(continued)

Supplemental Table 1
Continued

Variables	Type	Level	Included	Variable notes
Meets the WHO pupil to latrine ratio standards for girls	Observed	School level	Yes	This was derived from the pupil to latrine ratio variable
Meets the WHO pupil to latrine ratio standards for boys	Observed	School level	Yes	This was derived from the pupil to latrine ratio variable
Latrines clean at school	Observed	School level	No, redundant	These are intrinsically a latrine-level variables, but latrine-level analyses were not possible for our study. The correlation coefficient between these variables was −0.93 ($P < 0.01$). We used the visible feces variable as it was more relevant to the fecal-oral transmission mechanism. The variable was defined as the percentage of latrines in the school with no visible feces inside any of the latrines
Feces visible in latrines at school	Observed	School level	Yes	
Anal cleansing at school	Pupil reported	Pupil level	Yes	This variable was recategorized as a three-level variable (water vs. leaves/rocks/nothing vs. nothing) due to small cell counts
Home hygiene				
Have a handwashing place	Pupil reported	Pupil level	No, only adherence to handwashing is relevant	Handwashing only functions through adherence, so having access alone was not important to our models. We considered a Mokken scale for all of these variables, and they were highly scalable: Loevinger's H coefficient = 0.77.* However, the scale overemphasized access and underemphasized actual adherence. The correlation coefficient between last handwashing and always handwashing was 0.29 ($P < 0.01$). We used the always handwashing variable in our primary analysis as it represented the public health ideal, but we used the last handwashing variable in a sensitivity analysis as it is less prone to recall bias†
Water always available at that place	Pupil reported	Pupil level	No, only adherence to handwashing is relevant	
Soap always available at that place	Pupil reported	Pupil level	No, only adherence to handwashing is relevant	
Handwashed with soap and water the last time they defecated	Pupil reported	Pupil level	No, redundant	
Always handwash with soap and water after defecating	Pupil reported	Pupil level	Yes	
Home water				
Have an improved water source for drinking	Pupil reported	Pupil level	Yes	We used the improved water source variable. Pupils reported that water was generally available for drinking at home
Water always available for drinking	Pupil reported	Pupil level	No, homogenous	
Home sanitation				
Have personal toilet/latrine in home	Pupil reported	Pupil level	Yes	This variable was collected using two different questions and was later categorized into a single variable (personal vs. shared vs. none)
Have shared toilet/latrine in home	Pupil reported	Pupil level		
No toilet/latrine in home	Pupil reported	Pupil level		
Usually defecate in the latrine/toilet at home	Pupil reported	Pupil level	No, homogenous	These both measured a similar construct, but were very homogenous, so we did not use either of them
Used a latrine/toilet at home last time they defecated	Pupil reported	Pupil level	No, homogenous	
Anal cleansing at home	Pupil reported	Pupil level	Yes	This variable was recategorized as a three-level variable (water vs. leaves/rocks/nothing vs. nothing) due to small cell counts
Other WaSH variables				
Shoe wearing	Observed	Pupil level	Yes	This variable was observed, but may not reflect long-term shoe wearing behavior
Type of floor in home	Pupil reported	Pupil level	Yes	This variable was recategorized as a binary variable (earth/sand vs. cement/wood/iron sheets) due to small cell counts
Student eats soil (geophagy)	Pupil reported	Pupil level	Yes	This was a binary variable (yes vs. no), and may not reflect long-term practices

(continued)

Supplemental Table 1

Continued

Variables	Type	Level	Included	Variable notes
Confounders				
Grade	Pupil reported	Pupil level	Yes	Categorical variables, grades 2–6
Sex	Observed uniforms	Pupil level	Yes	Male vs. female
Siblings under age 5	Pupil reported	Pupil level	Yes	This count variable was recategorized as a binary variable (yes vs. no)
Wealth score	Pupil reported	Pupil level	Yes	This variable was derived from many different household asset variables, using principal component analysis[2]
Baseline *Ascaris lumbricoides* prevalence	Measured	Aggregated to school level	No	Pupil STH infection was measured at the baseline visit
Mean annual temperature	Measured	School level	Yes	Linked to school locations from http://www.worldclim.org/bioclim
Annual precipitation	Measured	School level	Yes	This was used as a proxy of geography. Former province was used instead of district because of the large number of districts in our study
Province	Observed	Province level	Yes	

STH = soil-transmitted helminth.

*Mokken suggested that a Loevinger's H coefficient of ≥ 0.5 denoted a strong scale.[3]

†We found similar results in unadjusted analyses that used the "last handwash" variables, instead of the "always handwash" variables. Pupils who reported washing their hands after they last defecated at school had an odds ratio (OR) of 0.75 (95% confidence interval [CI]: 0.54–1.06; $P = 0.10$), and for washing their hands after they last defecated at home the OR was 0.94 (95% CI: 0.69–1.28; $P = 0.68$).

‡The unadjusted analysis assessing the association between an improved water source at school and *A. lumbricoides* showed a stronger association (OR: 0.40, 95% CI: 0.18–0.87; $P = 0.02$) than the analysis that instead used the definition of an improved source that reliably supplied water (OR: 1.14, 95% CI: 0.58–2.21; $P = 0.071$).

SUPPLEMENTAL APPENDIX 2

Supplemental Table 2

Unadjusted ORs* comparing WaSH and *Ascaris lumbricoides*, *Trichuris trichiura*, and hookworm infection

	A. lumbricoides		*T. trichiura*		Hookworm	
	OR (95% CI)	*P*	OR (95% CI)	*P*	OR (95% CI)	*P*
School WaSH variables						
Always handwashed after defecation						
Yes	0.59 (0.35–1.01)	0.05	1.17 (0.53–2.55)	0.70	1.51 (0.67–3.4)	0.32
No	Referent		Referent		Referent	
Improved water source that reliably supplied water						
Yes	1.14 (0.58–2.21)	0.71	1.42 (0.47–4.36)	0.54	1.91 (0.66–5.57)	0.23
No	Referent		Referent		Referent	
Pupil to latrine ratio acceptable						
Yes	1.89 (1.22–2.91)	< 0.01	0.75 (0.32–1.74)	0.5	0.63 (0.18–2.23)	0.48
No	Referent		Referent		Referent	
Percent of latrines with visible feces on floor/walls						
All latrines have feces	1.26 (0.44–3.62)	0.67	1.24 (0.22–7.11)	0.81	0.23 (0.04–1.24)	0.09
No latrines have feces	Referent		Referent		Referent	
Percent of latrines that were VIP at school						
All latrines were VIP	0.45 (0.21–0.96)	0.04	0.84 (0.22–3.22)	0.8	0.99 (0.28–3.52)	0.98
No latrines were VIP	Referent		Referent		Referent	
Feces visible outside latrines						
Yes	1.48 (0.73–2.98)	0.28	1.36 (0.41–4.45)	0.62	1.23 (0.4–3.77)	0.72
No	Referent		Referent		Referent	
Anal cleansing with						
Water	1.04 (0.62–1.75)	0.89	0.85 (0.28–2.61)	0.77	1.93 (0.71–5.25)	0.20
Leaves/rocks/nothing	Referent		Referent		Referent	
Paper product	1.01 (0.80–1.28)	0.93	0.83 (0.54–1.27)	0.38	0.88 (0.46–1.67)	0.69
Home WaSH variables						
Always handwashed after defecation						
Yes	0.94 (0.69–1.28)	0.68	1.5 (0.89–2.52)	0.13	1.59 (0.89–2.82)	0.12
No	Referent		Referent		Referent	
Improved water source						
Yes	0.94 (0.77–1.16)	0.58	1.19 (0.82–1.74)	0.36	1.41 (0.87–2.28)	0.16
No	Referent		Referent		Referent	
Toilet						
Shared	1.06 (0.86–1.32)	0.58	1.09 (0.74–1.6)	0.67	Did not converge	
No toilet	0.99 (0.57–1.69)	0.96	0.70 (0.34–1.44)	0.33	Did not converge	
Personal	Referent		Referent		Referent	
Anal cleansing with						
Water	1.23 (0.77–1.98)	0.38	0.81 (0.28–2.33)	0.69	2.1 (0.94–4.71)	0.94
Leaves/rocks/nothing	Referent		Referent		Referent	
Paper product	0.91 (0.74–1.11)	0.34	1.01 (0.7–1.47)	0.28	0.76 (0.46–1.27)	0.46
Other WaSH variables						
Shoe wearing						
Closed shoes	0.60 (0.48–0.74)	< 0.01	0.73 (0.5–1.08)	0.12	0.41 (0.24–0.71)	0.24
Sandals	0.58 (0.45–0.74)	< 0.01	0.57 (0.36–0.9)	0.02	0.55 (0.3–1)	0.30
No shoes	Referent		Referent		Referent	
Type of floor in home						
Earth/sand	1.28 (1.02–1.62)	0.04	1.18 (0.79–1.77)	0.4	1.20 (0.62–2.34)	0.62
Cement/wood/iron sheets	Referent		Referent		Referent	
Student eats soil (geophagy)†						
Yes	1.14 (0.82–1.58)	0.45	0.51 (0.26–0.98)	0.04	1.69 (0.79–3.61)	0.79
No	Referent		Referent		Referent	

CI = confidence intervals; OR = odds ratio; WaSH = water, sanitation, and hygiene; VIP = ventilated improved pit latrine.
*Models accounted for clustering of pupils within schools.
†A soil eating practice common in Kenya.[29]

SUPPLEMENTAL APPENDIX 3

Supplemental Table 3

ORs showing interaction between pupil handwashing and type of water source among 4,404 pupils attending 51 Kenyan primary schools

	Improved water source†	Unimproved water source	
	OR (95% CI)	OR (95% CI)	*P* assessing interaction
Sensitivity analysis model 1*			
Last handwashed at school†	0.66 (0.43–1.03); *P* = 0.07	1.06 (0.58–1.91); *P* = 0.86	0.21
Last handwashed at home	0.98 (0.72–1.33); *P* = 0.90	1.46 (1.10–1.92); *P* < 0.01	0.05
	Improved water source (JMP definition)	Unimproved water source	*P* assessing interaction
Sensitivity analysis model 2*			
Always handwash at school	0.58 (0.32–1.05); *P* = 0.07	4.38 (0.66–28.93); *P* = 0.12	0.04
Always handwash at home	0.82 (0.50–1.32); *P* = 0.41	1.15 (0.73–1.80); *P* = 0.54	0.29
Sensitivity analysis model 3*			
Last handwashed at school	0.70 (0.47–1.05); *P* = 0.08	1.20 (0.55–2.60); *P* = 0.65	0.22
Last handwashed at home	0.99 (0.72–1.34); *P* = 0.94	1.45 (1.11–1.92); *P* < 0.01	0.05

CI = confidence interval; JMP = joint monitoring program; OR = odds ratio.
*Models included handwashing × water interaction terms and controlled for all of the other water, sanitation, and hygiene (WaSH) variables and confounder variables.
†At many schools, improved school water sources did not reliably supply water throughout the year, so here we constrained the definition of an improved school water source to also require water reliability.
